# Whole-Genome Sequence of Toxic Freshwater Cyanobacterium Chrysosporum ovalisporum Strain UAM-MAO

**DOI:** 10.1128/MRA.00819-18

**Published:** 2018-10-11

**Authors:** Soledad Sanz-Alférez, Carolina E. Rodríguez-Sanz, Ángel Barón-Sola, Francisca F. del Campo

**Affiliations:** a Departamento de Biología, Universidad Autónoma de Madrid, Madrid, Spain; Indiana University Bloomington

## Abstract

Here, we report the complete nucleotide sequence of *Chrysosporum ovalisporum* UAM-MAO, a filamentous, cylindrospermopsin-producing cyanobacterium involved in bloom forming in freshwater systems worldwide. It was isolated from an artificial pond in Madrid, Spain. The genome sequence contains 336 contigs, consisting of 7,478,035 bp and 2,851 putative protein-coding genes.

## Abstract (precis)

Here, we report the complete nucleotide sequence of *Chrysosporum ovalisporum* UAM-MAO, a filamentous, cylindrospermopsin-producing cyanobacterium involved in bloom forming in freshwater systems worldwide. It was isolated from an artificial pond in Madrid, Spain.

## ANNOUNCEMENT

The development of toxic harmful algal blooms is frequently correlated with climate change and eutrophication, in which cyanobacteria are significant, since a high concentration of nitrogen and phosphorous contribute to the massive proliferation of toxic cyanobacteria in water reservoirs (1). Cyanobacteria are organisms producing a great number of secondary metabolites with biological activity, including toxic products denominated cyanotoxins, which are common pollutants in freshwater systems. Among them, cylindrospermopsin (CYN), a potent alkaloid and protein synthesis inhibitor, is of increasing concern due to the growing number of detections reported worldwide in the last few years (2). Various cyanobacterium species have been identified as CYN producers, and Chrysosporum ovalisporum is one of them. This bacterium has an invasive behavior and is becoming an important health hazard because most strains are toxic (3). The gene clusters (*cyr* and *aoa*) involved in CYN synthesis have been completely described in several cyanobacteria (4, 5), showing several rearrangements in gene order and different flanking regions.

Chrysosporum ovalisporum (formerly Aphanizomenon ovalisporum) strain UAM-MAO was isolated from an artificial pond in Juan Carlos Park, Madrid, Spain, during a bloom formation. The production of CYN has been detected, and the *aoaA*, *aoaB*, and *aoaC* gene sequences and expression have been characterized (6, 7). DNA was extracted following mechanical disruption in cetyltrimethylammonium bromide (CTAB) buffer and treatment with proteinase K and lysozyme. A MiSeq paired-end genomic library was prepared and sequenced on an Illumina MiSeq platform (Parque Científico de Madrid, Spain). The reads were processed by Prinseq, and a *de novo* assembly was performed using SPAdes (8). Complementary metrics were examined by applying QUAST (9) to complete the annotation of the full genome using the BG7 system (10). Bioinformatic analysis revealed that the genome of UAM-MAO is approximately 7.47 Mbp in size, distributed in 336 contigs (≥1,000 bp), with a GC content of 50.39%. The annotation identified 2,851 coding sequences. Furthermore, studies of the UAM-MAO *cyr* gene cluster have been done, bearing a nucleotide sequence (up to 96% identity) similar to those of to Aphanizomenon sp. strain 10E6 (4) and Rhaphidiopsis curvata (5), but their genes are arranged in a different manner. In addition, secondary metabolites and other toxin biosynthesis genes were predicted by antiSMASH (11) using nonribosomal peptide synthetase (NRPS) and/or polyketide synthase (PKS) gene identification.

The availability of this genome may allow for a greater understanding of gene diversity and evolution within cyanobacterium organisms; also, it will improve our knowledge of the *cyr* cluster gene organization, as well as help to predict the regulation of cyanotoxins and secondary metabolite biosynthesis.

### Data availability.

This whole-genome shotgun project has been deposited at DDBJ/ENA/GenBank under the accession no. CDHJ00000000. The version described in this paper is the first version, CDHJ01000000.
